# Statin Use Is Associated with Reduced Mortality in Patients with Interstitial Lung Disease

**DOI:** 10.1371/journal.pone.0140571

**Published:** 2015-10-16

**Authors:** Signe Vedel-Krogh, Sune F. Nielsen, Børge G. Nordestgaard

**Affiliations:** Department of Clinical Biochemistry, Herlev and Gentofte Hospitals, Copenhagen University Hospital, Herlev, Denmark; Helmholtz Zentrum München, GERMANY

## Abstract

**Introduction:**

We hypothesized that statin use begun before the diagnosis of interstitial lung disease is associated with reduced mortality.

**Methods:**

We studied all patients diagnosed with interstitial lung disease in the entire Danish population from 1995 through 2009, comparing statin use versus no statin use in a nested 1:2 matched study.

**Results:**

The cumulative survival as a function of follow-up time from the date of diagnosis of interstitial lung disease (n = 1,786+3,572) and idiopathic lung fibrosis (n = 261+522) was higher for statin users versus never users (log-rank: P = 7·10^−9^ and P = 0.05). The median survival time in patients with interstitial lung disease was 3.3 years in statin users and 2.1 years in never users. Corresponding values in patients with idiopathic lung fibrosis were 3.4 versus 2.4 years. After multivariable adjustment, the hazard ratio for all-cause mortality for statin users versus never users was 0.73 (95% confidence interval, 0.68 to 0.79) in patients with interstitial lung disease and 0.76 (0.62 to 0.93) in patients with idiopathic lung fibrosis. Results were robust in all sensitivity analyses.

**Conclusion:**

Among patients with interstitial lung disease statin use was associated with reduced all-cause mortality.

## Introduction

Interstitial lung disease is a heterogeneous group of more than 300 different conditions and results from damage to lung parenchyma by various patterns of fibrosis and inflammation. There are four major forms of interstitial lung disease: Interstitial lung disease of known aetiology, idiopathic interstitial pneumonias, interstitial lung disease due to granulomatous disease, and others [1]. Idiopathic pulmonary fibrosis is the most common form of the idiopathic interstitial pneumonias, but the idiopathic forms include a variety of other diseases with very different clinical presentations and courses ranging from cryptogenic organising pneumonia with a very good prognosis to idiopathic pulmonary fibrosis with a median survival of less than 3 years [2]. Interstitial lung disease therapy includes anti-inflammatory and immunosuppressive therapies, with new anti-fibrotic agents introduced lately for the treatment of idiopathic pulmonary fibrosis [3].

Statins downregulate the mevalonate pathway limiting the synthesis of isoprenoid intermediates, which serve as lipid attachments for different intracellular signalling molecules [4], and thereby statins inhibit the activation of the guanosine-5'-triphosphate (GTP) binding protein rat sarcoma (Ras) [5]. Such inactivation of Ras might lead to reduced fibroblast activity, reduced synthesis of collagen, and increased fibroblast apoptosis [6]. Limited synthesis of isoprenoid intermediates also includes inactivation of another Ras-like protein, the Ras homolog gene family member A (RhoA), which has a role in the regulation of fibroblast proliferation [7]. Finally, statins may have anti-inflammatory properties as statins reduce levels of C-reactive protein [8] as well as levels of proinflammatory cytokines and chemokines [9]. Consequently, the use of statins among patients with interstitial lung disease could inhibit fibroblast activity and reduce inflammation in the lungs, potentially leading to retardation of disease progression and ultimately to reduced mortality, although some studies claim the opposite [10–12]. Furthermore, it is an ongoing debate whether statins can induce interstitial lung disease.

Due to the potentially beneficial effect of statins on both fibrosis and inflammation, we hypothesized that statin use in patients with interstitial lung disease is associated with reduced mortality. To test this hypothesis, we studied all patients with interstitial lung disease in the entire Danish population from 1995 through 2009, comparing statin users versus never users.

## Methods

### Study population and interstitial lung disease

The national Danish Civil Registration System records all births, immigrations, emigrations and deaths in Denmark through the civil registration number, which is unique to every person living in Denmark and includes information on age and sex. The national Danish Civil Registration System is 100% complete, that is, for practical purposes no persons are lost to follow-up [13].

Persons with interstitial lung disease including idiopathic lung fibrosis diagnosed from January 1, 1995 through December 31, 2009 were identified using the national Danish Patient Registry [14], which is blinded to statin use and which records all discharge diagnosis from Danish hospitals including outpatients, using the unique civil registration number. Diagnoses was chosen as previously done [15], interstitial lung disease was the International Classification of Diseases, 10^th^ Revision codes J60-70 and J84, while the subgroup idiopathic lung fibrosis was J84.1. Please see S1 Table for more information regarding diagnoses.

### Statin use

The national Danish Registry of Medicinal Products Statistics records information on all prescribed drugs dispensed at Danish pharmacies from 1995 onwards, and is blinded to the interstitial lung disease diagnosis. Statins were classified according to the Anatomical Therapeutically Classification of Drugs code C10AA.

To ascertain interstitial lung disease patients with continuous and stable use of statin, we defined statin users as those who obtained a prescription within 6 months before, together with another prescription of statin within two years before the date of the interstitial lung disease diagnosis, as done previously [16]. All remaining interstitial lung disease patients with other patterns of statin use before the diagnosis were defined as irregular statin users.

To avoid reverse causation, statin use after the interstitial lung disease diagnosis was not included in the statistical analysis. However, statin use before the diagnosis of interstitial lung disease was used to indicate statin use before and after the interstitial lung disease diagnosis.

Because use of statins has been increasing from 1995 through 2009, we conducted a nested 1:2 matched study (that is, a study that matched each statin user with two interstitial lung disease patients who had never used statins) matching on sex, diagnostic code (idiopathic lung fibrosis versus other), age at interstitial lung disease diagnosis, and year of interstitial lung disease diagnosis. Such matching also allows for different diagnostic criteria and treatments of interstitial lung disease over time.

### All-cause and cause-specific mortality

The national Danish Civil Registration System records date of death from any cause, and we collected deaths through December 31^st^, 2011 allowing a minimum of 2 years follow-up for any identified patient with interstitial lung disease. For all deaths in Denmark, the national Danish Causes of Death Registry [17] additionally records information on date of death together with cause of death, also using the unique civil registration number. Causes of death are ranked in three as reported by physicians at hospitals, in general practice, and in forensic or pathology departments. Diagnosis listed as causes of death are classified according to the International Classification of Diseases 10^th^ edition (ICD-10). Cause-specific mortality was determined by the first of three ranked causes of death. Respiratory disease was ICD-10 codes J00-J998, cardiovascular disease was codes I00-I99, and the remainder was other causes.

### Chronic obstructive pulmonary disease, cardiovascular disease and diabetes mellitus

Diagnosis of chronic obstructive pulmonary disease, cardiovascular disease, and diabetes mellitus before the interstitial lung disease diagnosis were also identified using the national Danish Patient Registry, according to the ICD-10 codes J41-J44, I00-I99 and E10-E14, respectively. Prior chronic obstructive pulmonary disease, cardiovascular disease, and diabetes mellitus were included as confounders as they could be associated with statin use and risk of all-cause mortality. Also, there could be diagnostic overlap between ILD and chronic obstructive pulmonary disease.

Information on any treatment of idiopathic lung fibrosis and prior treatment of chronic obstructive pulmonary disease was also obtained from the national Danish Registry of Medicinal Products Statistics. Treatment of interstitial lung disease included azathioprine, N-acetylcystein, colchicine, systemic corticosteroids, cyclophosphamide, methotrexat, thalidomide, and mycophenolate, Anatomical Therapeutically Classification of Drugs code L04AX01, R05CB01, M04AC01, H02AB, L01AA01, L04AX03, L04AX02, and L04AA06, respectively. Treatment of chronic obstructive pulmonary disease included beta2-adrenergic agonists, anticholinergic medication, theophylline, and inhaled glucocorticoids. Anatomical Therapeutically Classification of Drugs code R03AC, R03BB, R03DA, and R03BA, respectively.

### Other covariates

Statistics Denmark gathers information concerning ethnic descent, highest obtained level of education, and geographical residence of all persons living in Denmark.

### Propensity score

In order to address unknown patterns and other potential biases, such as healthy user bias, between statin users and never users, propensity score analysis was applied as done previously [16]. The propensity score was calculated using treatment with vitamin K antagonists as well as treatment of hypertension and depression as a proxy for regularly visiting a doctor’s office, thereby increasing a person’s chance of starting treatment with statins. Information on such treatment was obtained from the national Danish Registry of Medicinal Products Statistics. We collected information on vitamin K antagonists (B01AA) as well as diuretics and calcium antagonists (classified according to the Anatomical Therapeutically Classification of Drugs code C08DA, C08CA, C08DB, C03AA, C03AB, C03BA, C03CA, C093CB, C03DA, C03EA), angiotensin-converting-enzyme inhibitors and angiotensin II receptor blockers (C09AB, C09BA, C09BB, C08DA51, C09CA, C09DA, C09DB01), beta 1 antagonists (C07AA, C07AB, C07BB, C07CB, C07AG01, C07AB02) and antidepressants (N06).

### Statistical analysis

We used STATA 13.0 MP software. Interstitial lung disease patients below 40 years of age were excluded, as these patients are unlikely to receive statins. We conducted a nested 1:2 matched study by selecting exactly two random never users for each statin user matching on sex, diagnostic code (idiopathic lung fibrosis versus other), age at interstitial lung disease diagnosis, and year of interstitial lung disease diagnosis.

Cumulative survival curves using Kaplan-Meier estimates were compared with log-rank tests. Cox regression models using time after the interstitial lung disease diagnosis as time scale were used to calculate hazard ratios with 95% confidence intervals. Cox models were multivariable adjusted for sex (matching), diagnostic code (matching) year of birth (matching), age at diagnosis (matching), chronic obstructive pulmonary disease before interstitial lung disease, cardiovascular disease before interstitial lung disease, diabetes mellitus before interstitial lung disease, chronic obstructive pulmonary disease medication before interstitial lung disease, any medication for interstitial lung disease, descent (96% Danish versus other), highest obtained level of education, and geographical residency. The missing indicator method was used to account for missing information for education (5.8%) [18]. Propensity score was calculated using a logistic regression model and the score was included in the Cox model as an additional adjustment.

Competing risk models were calculated using the method of Fine and Gray and the calculation of subhazard ratios for cause-specific mortality. Furthermore, we stratified on sex, prior chronic obstructive pulmonary disease, prior cardiovascular disease, prior diabetes mellitus, geographical residency, education, year of interstitial lung disease diagnosis, and age at diagnosis.

All 5,358 interstitial lung disease patients were followed from date of diagnosis and censored at the date of death (n = 3,266), emigration (n = 7), or December 31, 2011 (n = 2,085), whichever came first. Likewise, all 783 idiopathic lung fibrosis patients were censored at death (n = 537), emigration (n = 1), or December 31^st^, 2011 (n = 245).

### Ethics statement

Ethics committee approval is not necessary for an anonymous registry based epidemiology study in Denmark.

## Results

### Study population

**Fig 1:**
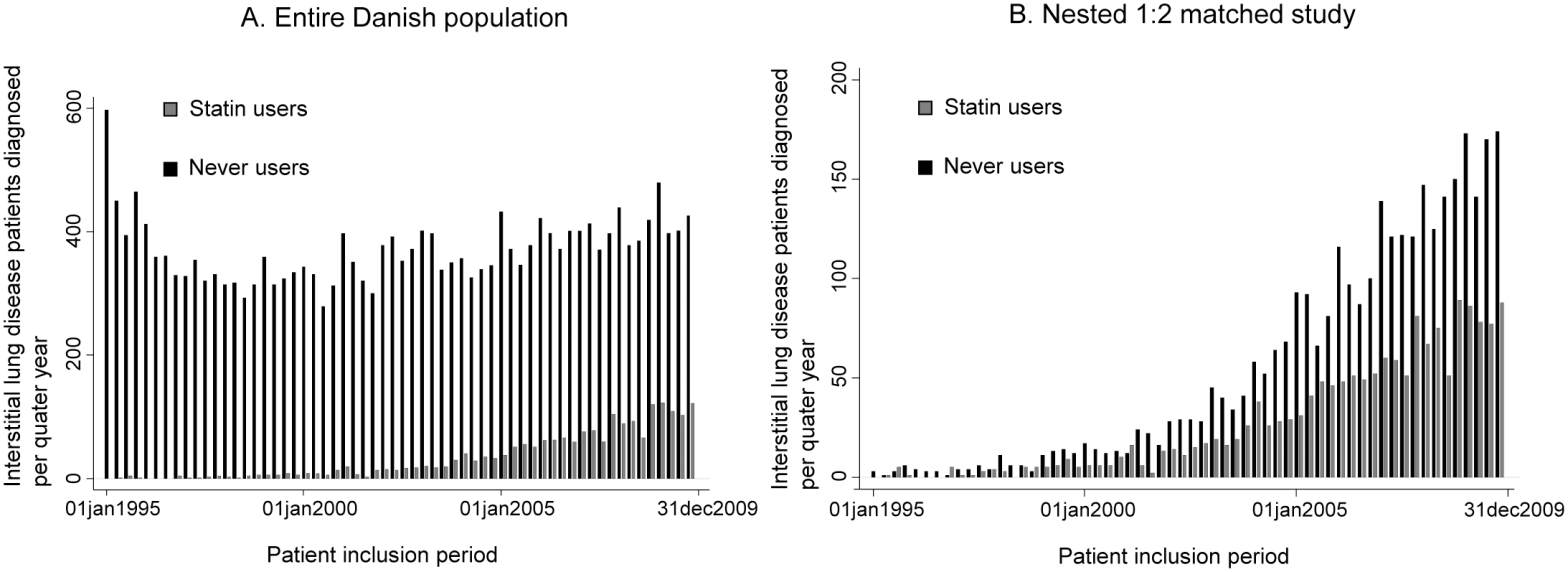
Inclusion of interstitial lung disease patients during 1995 through 2009. Patients were divided into regular statin users and never users. A, Entire Danish population. B. Nested 1:2 matched study.

**Fig 2:**
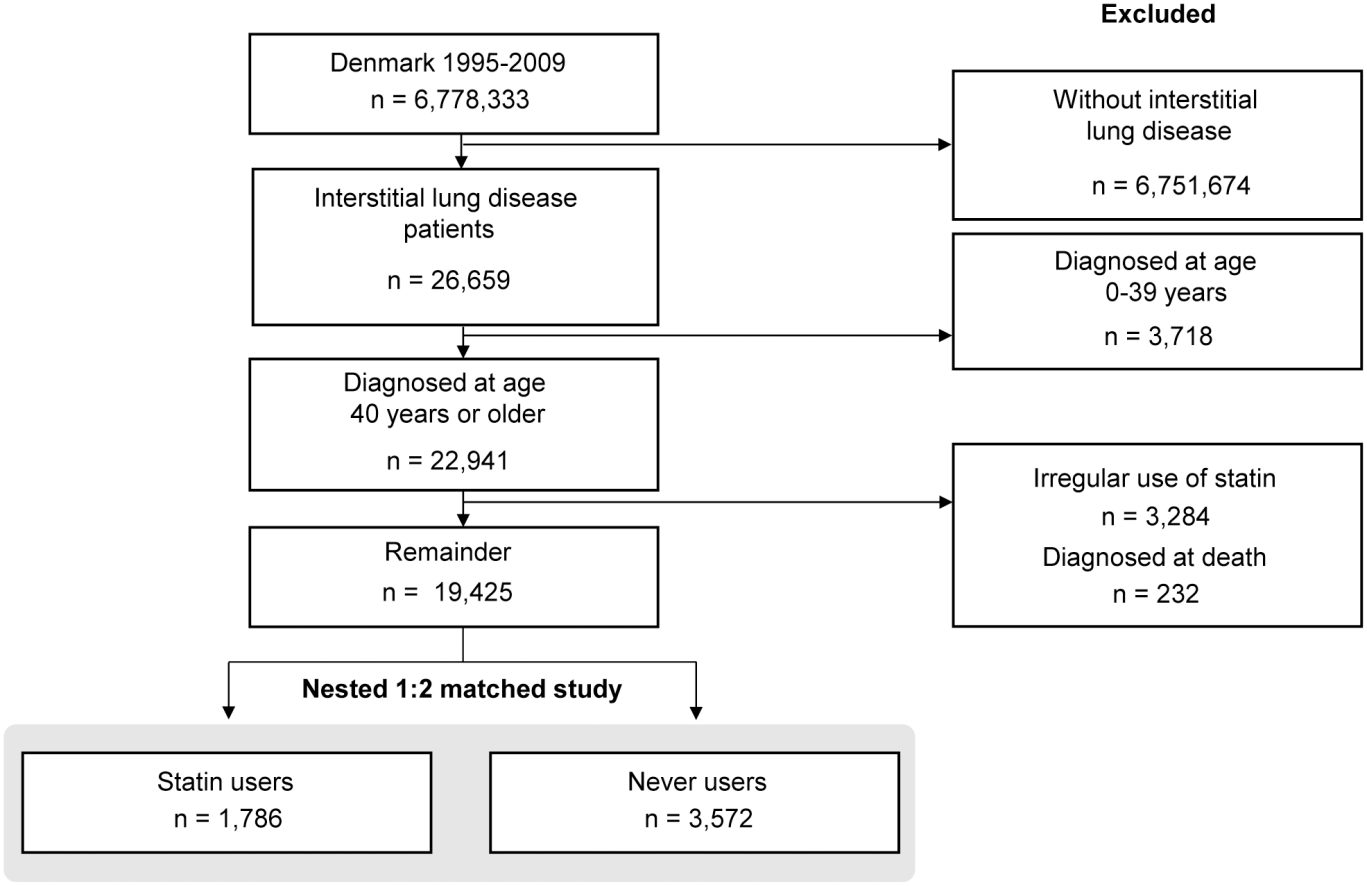
Selection of interstitial lung disease patients entering into the study. Patients were divided into regular statin users and never users for the nested 1:2 matched study.

We included patients from the entire Danish population aged 40 years or older who had received a diagnosis of interstitial lung disease between 1995 and 2009, and followed them until December 31, 2011 (median, 2.5 years; range, 0 to 16.5); we identified a total of 22,941 patients with interstitial lung disease aged 40 or older, of whom 5,915 had idiopathic lung fibrosis. By including only patients who received a diagnosis of interstitial lung disease through 2009, we allowed at least 2 years of follow-up time for all patients. Due to an increase in the use of statins (Fig 1) and possible changes in diagnosis and treatment of interstitial lung disease over time, we conducted a nested 1:2 matched study. In the matched study, we grouped 1,786 as regular statin users up until the time of the interstitial lung disease diagnosis, whereas 3,572 had never used statins (Fig 2).

Baseline characteristics of these patients are shown in Table 1. During 13,580 person-years of follow-up, 3,266 patients died: 812 from respiratory diseases, 749 from cardiovascular diseases, and 1,705 from other causes.

**Table 1:** Baseline characteristics of patients with interstitial lung disease and idiopathic lung fibrosis diagnosed at age 40 or older and followed from 1995 through 2011.

	Interstitial lung disease			Idiopathic lung fibrosis		
	Statin users	Never users	P value	Statin users	Never users	P value
**Number**	1,786	3,572		261	522	
**Age at diagnosis, years**	71 (64–78)	71 (64–78)	0.96	73 (67–78)	73 (67–78)	0.96
**Sex**			1.00			1.00
Female	646 (36%)	1,292 (36%)		99 (38%)	198 (38%)	
Male	1,140 (64%)	2,280 (64%)		162 (62%)	324 (62%)	
**Any interstitial lung disease treatment**			0.05			0.46
No	657 (37%)	1,141 (40%)		41 (16%)	93 (18%)	
Yes	1,129 (63%)	2,158 (60%)		220 (84%)	429 (82%)	
**COPD diagnosis before interstitial lung disease**			0.30			0.03
No	1,361 (76%)	2,767 (77%)		185 (71%)	328 (63%)	
Yes	425 (24%)	805 (23%)		76 (57%)	194 (37%)	
**COPD treatment before interstitial lung disease**			0.08			0.45
No	1,174 (66%)	2,432 (68%)		149 (57%)	283 (54%)	
Yes	612 (34%)	1,140 (32%)		112 (43%)	239 (46%)	
**Cardiovascular disease before interstitial lung disease**			2∙10^−128^			1∙10^−19^
No	279 (16%)	1,771 (50%)		45 (17%)	266 (51%)	
Yes	1,507 (84%)	1,801 (50%)		216 (83%)	256 (49%)	
**Diabetes mellitus before interstitial lung disease**			1∙10^−84^			2∙10^−15^
No	1,334 (75%)	3,341 (94%)		196 (75%)	494 (95%)	
Yes	452 (25%)	231 (6%)		65 (25%)	28 (5%)	
**Residential city size**			0.97			0.41
<12,000 or rural	666 (37%)	1,314 (37%)		99 (38%)	196 (38%)	
12,000–100,000	423 (24%)	879 (25%)		85 (33%)	146 (28%)	
>100,000	697 (39%)	1,379 (38%)		77 (34%)	180 (34%)	
**Level of education**			0.23			0.47
Not available	98 (5%)	212 (6%)		14 (5%)	31 (6%)	
Primary or high school	843 (48%)	1,626 (46%)		129 (49%)	263 (51%)	
Vocational	624 (35%)	1,203 (34%)		80 (31%)	163 (31%)	
Academic	221 (12%)	531 (14%)		38 (15%)	65 (12%)	

Data are n (%) or median (interquartile range). Baseline characteristics were at the date of diagnosis of interstitial lung disease and idiopathic lung fibrosis. The nested 1:2 matched study was matched on sex, diagnostic code (idiopathic lung fibrosis versus other), age at diagnosis, and year of diagnosis. Only interstitial lung disease/ idiopathic lung fibrosis patients using statins with two exact matching controls were included. Any interstitial lung disease treatment (azathioprine, N-acetylcystein, colchicine, systemic corticosteroids, cyclophosphamide, methotrexat, thalidomide, and mycophenolate) and chronic obstructive pulmonary disease treatment (beta2-adrenergic agonists, anticholinergic medication, theophylline, and inhaled glucocorticoids) were available from 1995–2011. Residential city size designates the location for the longest period of residence. Level of education is the highest obtained level.

### All-cause mortality

**Fig 3:**
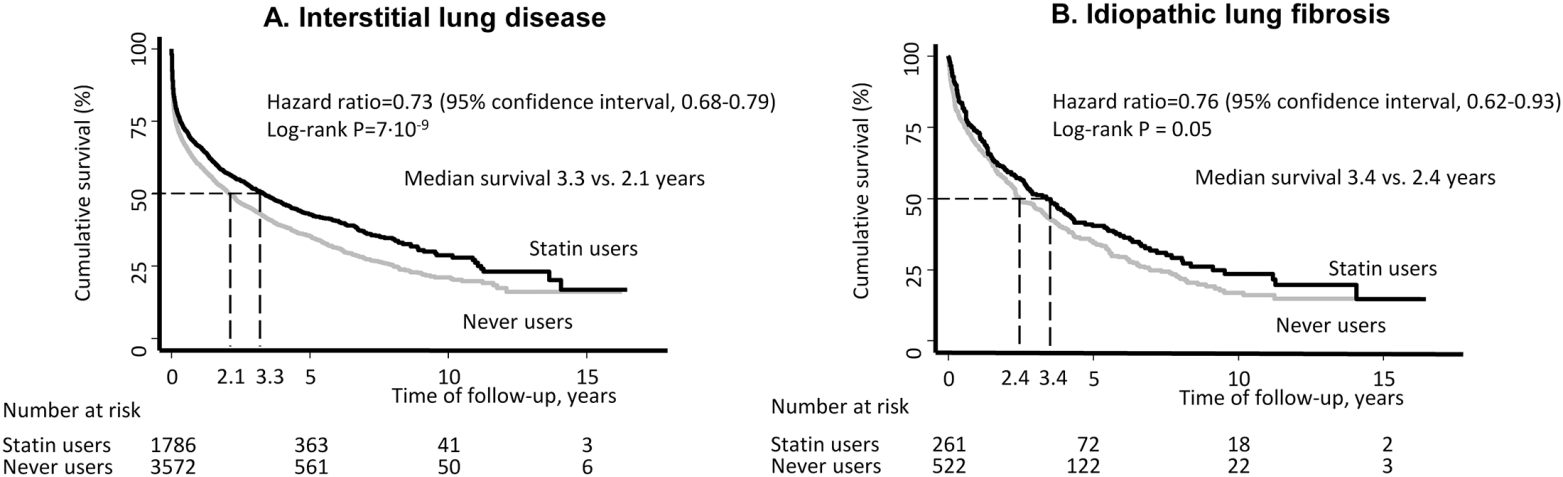
Survival and risk of all-cause mortality in statin users versus never users. Patients with interstitial lung disease (A) and patients with idiopathic lung fibrosis (B). Hazard ratios are shown after multivariable adjustments.

The cumulative survival as a function of follow-up time from the date of diagnosis of interstitial lung disease and idiopathic lung fibrosis was higher for statin users versus never users (log-rank: P = 7·10^−9^ and P = 0.05) (Fig 3). The median survival time in patients with interstitial lung disease was 3.3 years in statin users versus 2.1 years in never users. Corresponding values in patients with idiopathic lung fibrosis were 3.4 and 2.4 years. After multivariable adjustment the hazard ratio for all-cause mortality for statin users versus never users was 0.73 (95% confidence interval, 0.68 to 0.79) for interstitial lung disease and 0.76 (0.62 to 0.93) for idiopathic lung fibrosis.

### Cause-specific mortality

**Fig 4:**
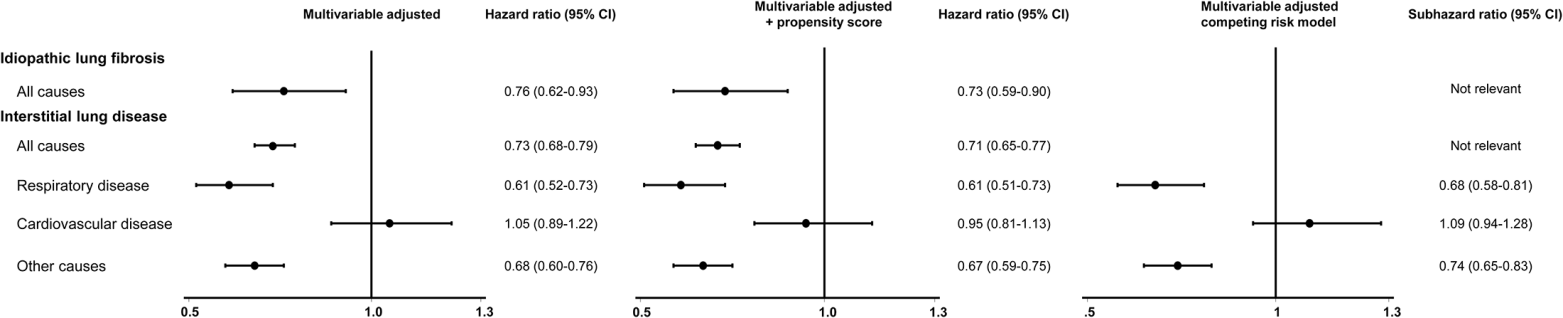
Risk of all-cause and cause-specific mortality in statin users versus never users. Hazard ratios are shown after multivariable adjustment, after additional adjustment for propensity score, and after multivariable adjustment with the use of Fine and Gray subhazard regression allowing for competing risk of death.

After multivariable adjustment, the hazard ratio for mortality for statin users versus never users from respiratory disease was 0.61 (95% CI, 0.52 to 0.73), for mortality from cardiovascular disease 1.05 (0.89–1.22), and for mortality from other causes 0.68 (0.60–0.76) (Fig 4). Among patients diagnosed with idiopathic lung fibrosis, the statistical power was too low to estimate reliable hazard ratios for cause-specific mortality.

### Sensitivity analyses

Attempting to adjust for different health patterns of the medical history of the interstitial lung disease patients studied, and thereby trying to avoid healthy user bias, we adjusted for propensity score: the results for all-cause mortality and cause-specific mortality remained similar (Fig 4). The results for cause-specific mortality also remained similar when we accounted for competing risk of death from other causes, with the use of Fine and Gray subhazard regression (Fig 4).

**Fig 5:**
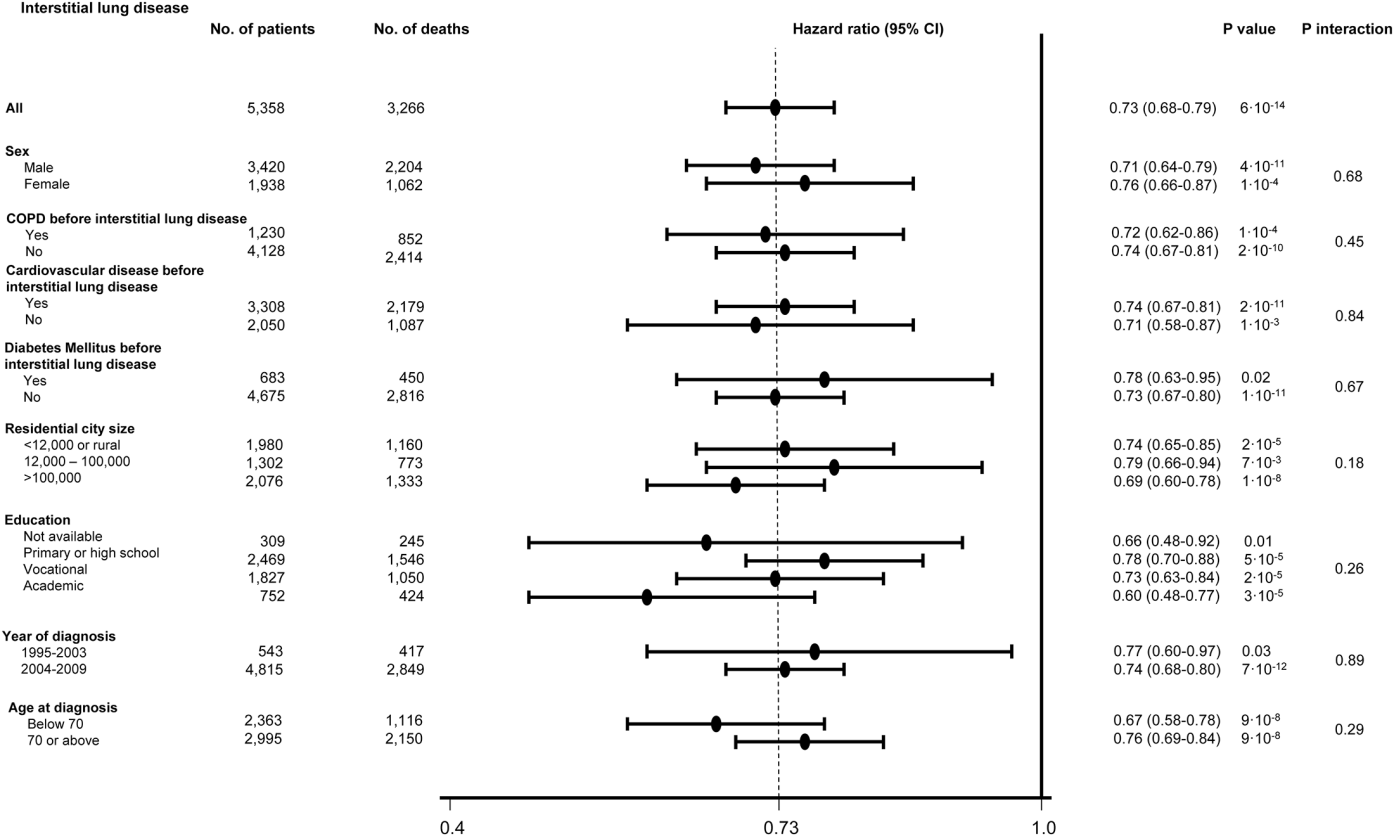
Risk of all-cause mortality in statin users versus never users stratified for covariates. Hazard ratios are shown after multivariable adjustments. Year of diagnosis was divided into two periods, 1995–2003 and 2004–2009, as a consensus on classification of idiopathic lung fibrosis was published in 2002 [19].

In analyses stratified for characteristics which could be associated with statin use, interstitial lung disease diagnosis, and/or risk of all-cause mortality, that is, for sex, chronic obstructive pulmonary disease before interstitial lung disease, cardiovascular disease before interstitial lung disease, diabetes mellitus before interstitial lung disease, residential city size, education, year of diagnosis and age at diagnosis, all-cause mortality was reduced in statin users versus never users in all strata (Fig 5).

Results for all-cause mortality were similar in sensitivity analyses when excluding individuals with ever-diagnosed chronic obstructive pulmonary disease (n = 1,880) from the interstitial lung disease population (S1 Fig), in a nested 1:2 matched study after excluding individuals diagnosed with aspiration pneumonia from the interstitial lung disease population (S2 Fig), when excluding individuals with ever-diagnosed rheumatic or connective tissue disease from the idiopathic lung fibrosis population (S3 Fig), and in a 1:2 nested matched study including individuals below 40 years of age (S4 Fig).

## Discussion

In this nested 1:2 matched nationwide study we observed that statin use was associated with reduced mortality in 5,358 patients with interstitial lung disease and in 783 patients with idiopathic lung fibrosis. This observation is novel. Although a cohort study such as ours cannot demonstrate causality, it is clinically reassuring that statin use do not seem to increase mortality among these patients. In accordance with this interpretation, a previous study with 35 statin users and 443 never users found no association between statin use and mortality in patients with idiopathic pulmonary fibrosis [20].

Mechanistically, statins inhibit cholesterol synthesis through inhibition of the rate limiting enzyme in the mevalonate and cholesterol synthesis pathway, 3-hydroxy-3-methylglutaryl coenzyme A (HMG-CoA) [4], but several studies have detected effects of statins beyond changing the lipid profile alone. Thus, statins may have anti-inflammatory effects as they reduce the level of C-reactive protein [8,21] and several other inflammatory mediators like tumor necrosis factor-α, fibrinogen, transforming growth factor-β and interleukins [9,22,23]; part of the reduction in C-reactive protein may be caused by statins ability to reduce plasma triglycerides and remnant cholesterol levels [24]. Furthermore, statins inhibit the synthesis of isoprenoid intermediates, which serve as lipid attachments for a variety of intracellular signalling molecules, among others the ρ family of intracellular guanosine triphosphat binding proteins, which in the absence of statins can mediate a proinflammatory respons [25]. Inhibition of isoprenoid synthesis might also disrupt cellular growth, secretion and signal transduction [26], and thus statins may affect activation and proliferation of many cells involved in lung inflammation. Accordingly, anti-inflammatory effects of statins could reduce the inflammation in the lungs of patients with interstitial lung disease, thus leading to reduced disease progression and to reduced mortality, as observed in the present study.

Not only inflammation, but also lung fibrosis is a hallmark of interstitial lung disease. Traditionally, it was believed that inflammation initiated fibrosis, but recently a study suggested that fibrosis precedes inflammation and develops as aberrant epithelial and epithelial–mesenchymal responses to chronic alveolar epithelial injury [27]. Importantly, statins may reduce fibrosis by lowering the profibrogenic cytokine transforming growth factor-β1 [26], and cell proliferation, collagen deposition, angiogenesis and fibroblast differentiation into the profibrogenic myofibroblast phenotype which characterise idiopathic pulmonary fibrosis, are all mediated through connective tissue growth factor induced by the cytokine transforming growth factor-β1. Also, statins reduce the tissue damage associated with cigarette smoking in rats [28] and rabbits [29], and in human macrophages [30] and monocytes [31] from smokers, probably by the reduction in matrix metalloproteinases-9 and airway remodelling. Furthermore, lovastatin may induce fibroblast apoptosis via Ras [6] and may reduce fibroblast proliferation via RhoA [7]. Taken together, the above mentioned findings suggest plausible mechanisms through reduced inflammation and fibrosis that might explain the presently observed reduced mortality among interstitial lung disease patients using statins.

Strengths of the present study include the large size of the study population. In this study we included all patients in Denmark diagnosed with interstitial lung disease from 1995 through 2009, and performed a nested 1:2 matched study to avoid influence from changes in statin use from 1995 through 2009.

Limitations of the present study might include selection bias, but as we included all patients with interstitial lung disease from the entire Danish population who were 40 years of age or older and eligible for statin use, without losses to follow-up, and as we performed a nested 1:2 matched study selection bias is unlikely to be a major problem. However, a potential limitation could be diagnosis misclassification. Interstitial lung disease is difficult to diagnose, the terminology has been poorly defined, especially for the idiopathic interstitial pneumonia diagnoses. The classification of interstitial lung disease has changed over time and this could bias our study. Only during the past 10 years has a consensus of classification been achieved [1,19,32]. Although idiopathic pulmonary fibrosis is the most common form of idiopathic interstitial pneumonias, the idiopathic forms also includes a variety of other diseases that have very different presentations and clinical courses. Interstitial lung disease is even broader and includes a more diverse spectrum of diseases. Though some studies have argued that statin could potentially induce a mild form of interstitial lung disease [11,12] disturbing the results, a recent study found no evidence of an association between statin use and the diagnosis [10]. Thus, it seems unlikely that nondifferential misclassification could explain the results found in this study. Likewise important, through matching statin users with never users on sex, diagnostic code, age at interstitial lung disease diagnosis, and year of diagnosis we secured that changes in diagnostic criteria and treatment of interstitial lung disease and idiopathic lung fibrosis from 1995 through 2011 are unlikely to explain our results.

The patients included in our study have been matched to live to an age where they were prescribed a regular statin treatment, thus this may be a selected patient group with a different survival time compared to all ILD patients; however, even if this was the case, this would not explain the present results. Mechanistically, statins could improve pulmonary hypertension and epidemiologic studies have claimed a benefit of statins in patients with this disease [33]. Nonetheless, clinical trials did not show any benefit of statins in patients with pulmonary arterial hypertension [34]. However, our study could not adjust for pulmonary hypertension, a complication of interstitial lung disease, including idiopathic lung fibrosis. Likewise, information on smoking status, obesity, and hypercholesterolemia could not be adjusted for in our analyses as this information is not available in the national Danish registers. Another concern is the possibility that statin users are not representative of the general population, but represents individuals who by taken statins also engage in a healthier lifestyle and see a primary care physician more often, thereby creating a healthy user effect which could potentially bias our study. However, when we adjusted for propensity score, which accounted for the patient’s probability of being treated with statin on the basis of patterns in the medical history, we found results similar to the main findings. Nevertheless, this approach can never fully exclude healthy user bias [35]. The magnitude of the effect of statins in this study seems large, and therefore we might not have excluded healthy user bias completely.

In conclusion, among patients diagnosed with interstitial lung disease we observed an association between statin use and reduced risk of mortality which is clinically reassuring on its own.

## Supporting Information

S1 Fig Survival and risk of all-cause mortality in statin users versus never users among individuals diagnosed with interstitial lung disease excluding individuals with ever diagnosed chronic obstructive pulmonary disease.
Hazard ratio is shown after multivariable adjustments.
(PDF)

S2 Fig Survival and risk of all-cause mortality in statin users versus never users in a nested 1:2 matched study excluding individuals with aspiration pneumonia from the interstitial lung disease population.
Hazard ratio is shown after multivariable adjustments.
(PDF)

S3 Fig Survival and risk of all-cause mortality in statin users versus never users among individuals diagnosed with idiopathic lung fibrosis excluding individuals with ever diagnosed rheumatic or connective tissue disease.
Hazard ratio is shown after multivariable adjustments.
(PDF)

S4 Fig Survival and risk of all-cause mortality in statin users versus never users in a nested 1:2 matched study with inclusion of patients below 40 years of age. Hazard ratios are shown after multivariable adjustments.
(PDF)

S1 Table Interstitial lung disease diagnosis codes among the patients included in the nested 1:2 matched study and from all such patients diagnosed in Denmark 1995–2009. (PDF)

## References

[1] Bradley B, Branley HM, Egan JJ, Greaves MS, Hansell DM, Harrison NK et al Interstitial lung disease guideline: the British Thoracic Society in collaboration with the Thoracic Society of Australia and New Zealand and the Irish Thoracic Society. Thorax. 2008;63 Suppl 5: v1–58. 10.1136/thx.2008.101691 18757459

[2] Bjoraker JA, Ryu JH, Edwin MK, Myers JL, Tazelaar HD, Schroeder DR et al Prognostic significance of histopathologic subsets in idiopathic pulmonary fibrosis. Am J Respir Crit Care Med. 1998;157: 199–203. 9445300 10.1164/ajrccm.157.1.9704130

[3] Lota HK, Wells AU. The evolving pharmacotherapy of pulmonary fibrosis. Expert Opin Pharmacother. 2013;14: 79–89. 10.1517/14656566.2013.758250 23265249

[4] Goldstein JL, Brown MS Regulation of the mevalonate pathway. Nature. 1990;343: 425–430. 1967820 10.1038/343425a0

[5] Van AL, D'Souza-Schorey C. Rho GTPases and signaling networks. Genes Dev. 1997;11: 2295–2322. 9308960 10.1101/gad.11.18.2295

[6] Tan A, Levrey H, Dahm C, Polunovsky VA, Rubins J, Bitterman PB. Lovastatin induces fibroblast apoptosis in vitro and in vivo. A possible therapy for fibroproliferative disorders. Am J Respir Crit Care Med. 1999;159: 220–227. 9872842 10.1164/ajrccm.159.1.9802104

[7] Watts KL, Cottrell E, Hoban PR, Spiteri MA. RhoA signaling modulates cyclin D1 expression in human lung fibroblasts; implications for idiopathic pulmonary fibrosis. Respir Res. 2006;7: 88 16776827 10.1186/1465-9921-7-88 PMC1513217

[8] Albert MA, Danielson E, Rifai N, Ridker PM. Effect of statin therapy on C-reactive protein levels: the pravastatin inflammation/CRP evaluation (PRINCE): a randomized trial and cohort study. JAMA. 2001;286: 64–70. 11434828 10.1001/jama.286.1.64

[9] Forrester JS, Libby P. The inflammation hypothesis and its potential relevance to statin therapy. Am J Cardiol. 2007;99: 732–738. 17317382 10.1016/j.amjcard.2006.09.125

[10] Saad N, Camus P, Suissa S, Ernst P. Statins and the risk of interstitial lung disease: a cohort study. Thorax. 2013;68: 361–364. 10.1136/thoraxjnl-2012-201823 23299962

[11] Xu JF, Washko GR, Nakahira K, Hatabu H, Patel AS, Fernandez IE et al Statins and pulmonary fibrosis: the potential role of NLRP3 inflammasome activation. Am J Respir Crit Care Med. 2012;185: 547–556. 10.1164/rccm.201108-1574OC 22246178 PMC3297101

[12] Fernandez AB, Karas RH, Alsheikh-Ali AA, Thompson PD. Statins and interstitial lung disease: a systematic review of the literature and of food and drug administration adverse event reports. Chest. 2008;134: 824–830. 10.1378/chest.08-0943 18689579

[13] Pedersen CB, Gotzsche H, Moller JO, Mortensen PB. The Danish Civil Registration System. A cohort of eight million persons. Dan Med Bull. 2006;53: 441–449. 17150149

[14] Andersen TF, Madsen M, Jorgensen J, Mellemkjoer L, Olsen JH. The Danish National Hospital Register. A valuable source of data for modern health sciences. Dan Med Bull. 1999;46: 263–268. 10421985

[15] Sode BF, Dahl M, Nielsen SF, Nordestgaard BG. Venous thromboembolism and risk of idiopathic interstitial pneumonia: a nationwide study. Am J Respir Crit Care Med. 2010;181: 1085–1092. 10.1164/rccm.200912-1951OC 20167844

[16] Nielsen SF, Nordestgaard BG, Bojesen SE. Statin use and reduced cancer-related mortality. N Engl J Med. 2012;367: 1792–1802. 10.1056/NEJMoa1201735 23134381

[17] Helweg-Larsen K. The Danish Register of Causes of Death. Scand J Public Health. 2011;39: 26–29. 10.1177/1403494811399958 21775346

[18] Donders AR, van der Heijden GJ, Stijnen T, Moons KG. Review: a gentle introduction to imputation of missing values. J Clin Epidemiol. 2006;59: 1087–1091. 16980149 10.1016/j.jclinepi.2006.01.014

[19] American Thoracic Society/European Respiratory Society International Multidisciplinary Consensus Classification of the Idiopathic Interstitial Pneumonias. This joint statement of the American Thoracic Society (ATS), and the European Respiratory Society (ERS) was adopted by the ATS board of directors, June 2001 and by the ERS Executive Committee, June 2001. Am J Respir Crit Care Med. 2002;165: 277–304. 11790668 10.1164/ajrccm.165.2.ats01

[20] Nadrous HF, Ryu JH, Douglas WW, Decker PA, Olson EJ. Impact of angiotensin-converting enzyme inhibitors and statins on survival in idiopathic pulmonary fibrosis. Chest. 2004;126: 438–446. 15302729 10.1378/chest.126.2.438

[21] Ridker PM, Danielson E, Fonseca FA, Genest J, Gotto AM Jr., Kastelein JJ et al Rosuvastatin to prevent vascular events in men and women with elevated C-reactive protein. N Engl J Med. 2008;359: 2195–2207. 10.1056/NEJMoa0807646 18997196

[22] Bu DX, Tarrio M, Grabie N, Zhang Y, Yamazaki H, Stavrakis G et al Statin-induced Kruppel-like factor 2 expression in human and mouse T cells reduces inflammatory and pathogenic responses. J Clin Invest. 2010;120: 1961–1970. 10.1172/JCI41384 20440076 PMC2877947

[23] Sadowitz B, Maier KG, Gahtan V. Basic science review: Statin therapy—Part I: The pleiotropic effects of statins in cardiovascular disease. Vasc Endovascular Surg. 2010;44: 241–251. 10.1177/1538574410362922 20403949

[24] Varbo A, Benn M, Tybjaerg-Hansen A, Nordestgaard BG. Elevated remnant cholesterol causes both low-grade inflammation and ischemic heart disease, whereas elevated low-density lipoprotein cholesterol causes ischemic heart disease without inflammation. Circulation. 2013;128: 1298–1309. 10.1161/CIRCULATIONAHA.113.003008 23926208

[25] Wang CY, Liu PY, Liao JK. Pleiotropic effects of statin therapy: molecular mechanisms and clinical results. Trends Mol Med. 2008;14: 37–44. 18068482 10.1016/j.molmed.2007.11.004 PMC2621332

[26] Hothersall E, McSharry C, Thomson NC. Potential therapeutic role for statins in respiratory disease. Thorax. 2006;61: 729–734. 16877692 10.1136/thx.2005.057976 PMC2104700

[27] Selman M, King TE, Pardo A. Idiopathic pulmonary fibrosis: prevailing and evolving hypotheses about its pathogenesis and implications for therapy. Ann Intern Med. 2001;134: 136–151. 11177318 10.7326/0003-4819-134-2-200101160-00015

[28] Lee JH, Lee DS, Kim EK, Choe KH, Oh YM, Shim TS et al Simvastatin inhibits cigarette smoking-induced emphysema and pulmonary hypertension in rat lungs. Am J Respir Crit Care Med. 2005;172: 987–993. 16002570 10.1164/rccm.200501-041OC

[29] Fukumoto Y, Libby P, Rabkin E, Hill CC, Enomoto M, Hirouchi Y et al Statins alter smooth muscle cell accumulation and collagen content in established atheroma of watanabe heritable hyperlipidemic rabbits. Circulation. 2001;103: 993–999. 11181475 10.1161/01.cir.103.7.993

[30] Bellosta S, Via D, Canavesi M, Pfister P, Fumagalli R, Paoletti R et al HMG-CoA reductase inhibitors reduce MMP-9 secretion by macrophages. Arterioscler Thromb Vasc Biol. 1998;18: 1671–1678. 9812903 10.1161/01.atv.18.11.1671

[31] Grip O, Janciauskiene S, Lindgren S. Atorvastatin activates PPAR-gamma and attenuates the inflammatory response in human monocytes. Inflamm Res. 2002;51: 58–62. 11926313 10.1007/BF02684000

[32] Ryu JH, Daniels CE, Hartman TE, Yi ES. Diagnosis of interstitial lung diseases. Mayo Clin Proc. 2007;82: 976–986. 17673067 10.4065/82.8.976

[33] Kao PN. Simvastatin treatment of pulmonary hypertension: an observational case series. Chest. 2005;127: 1446–1452. 15821229 10.1378/chest.127.4.1446

[34] Kawut SM, Bagiella E, Lederer DJ, Shimbo D, Horn EM, Roberts KE et al Randomized clinical trial of aspirin and simvastatin for pulmonary arterial hypertension: ASA-STAT. Circulation. 2011;123: 2985–2993. 10.1161/CIRCULATIONAHA.110.015693 21593252 PMC3427737

[35] Dormuth CR, Patrick AR, Shrank WH, Wright JM, Glynn RJ, Sutherland J et al Statin adherence and risk of accidents: a cautionary tale. Circulation. 2009;119: 2051–2057. 10.1161/CIRCULATIONAHA.108.824151 19349320 PMC2744446

