# Supplementary material for: Statin Use Is Associated with Reduced Mortality in Patients with Interstitial Lung Disease
Source: PLoS One. 2015 Oct 16;10(10):e0140571. doi: 10.1371/journal.pone.0140571 (PMC4608706; doi:10.1371/journal.pone.0140571)
Supplement: S1 Fig — Hazard ratio is shown after multivariable adjustments. (PDF) [file pone.0140571.s001.pdf]

S1 Figure. Survival and risk of all-cause mortality in statin users versus never users among individuals diagnosed with interstitial lung disease excluding individuals with ever diagnosed chronic obstructive pulmonary disease.

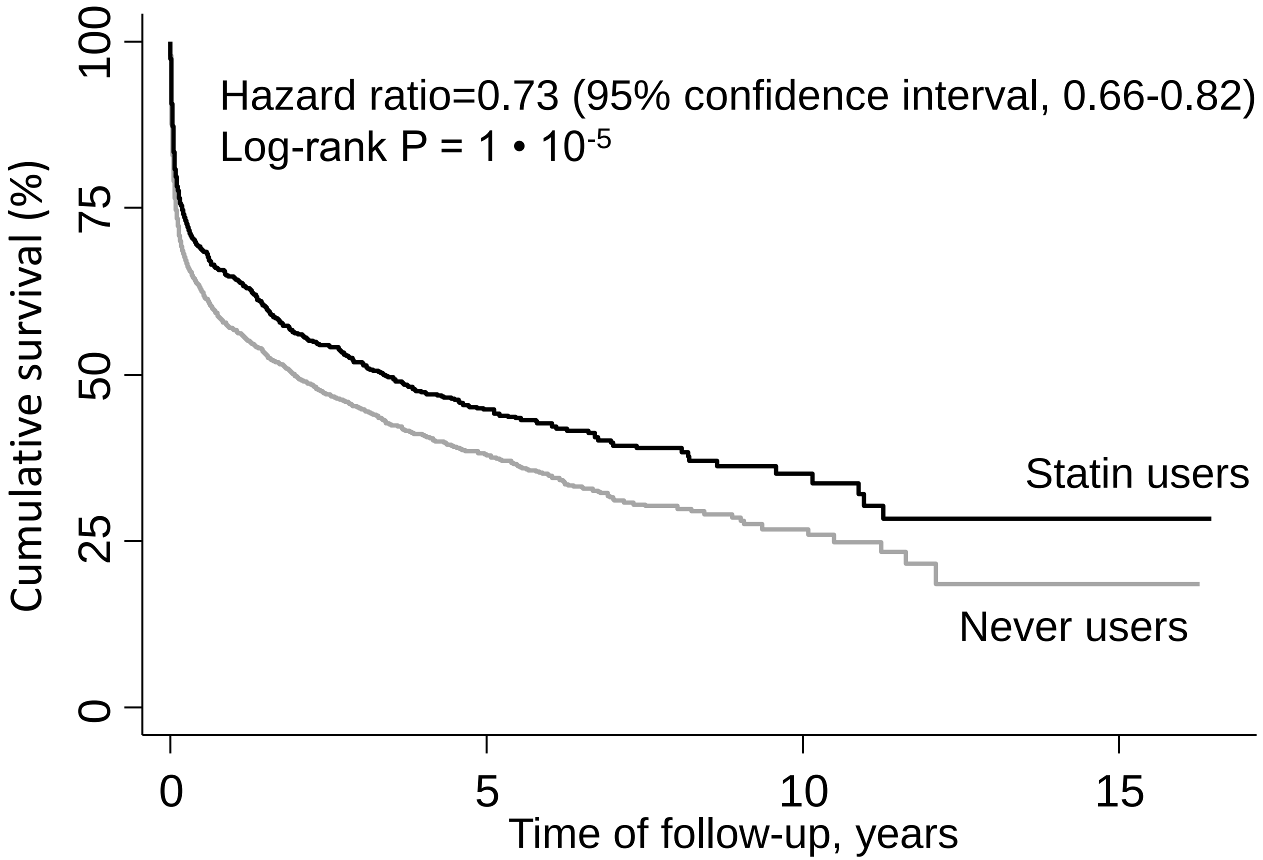

Number at risk				
Statin users	1,130	220	26	2
Never users	2,348	350	31	4
