# Supplementary material for: Statin Use Is Associated with Reduced Mortality in Patients with Interstitial Lung Disease
Source: PLoS One. 2015 Oct 16;10(10):e0140571. doi: 10.1371/journal.pone.0140571 (PMC4608706; doi:10.1371/journal.pone.0140571)
Supplement: S2 Fig — Hazard ratio is shown after multivariable adjustments. (PDF) [file pone.0140571.s002.pdf]

S2 Figure. Survival and risk of all-cause mortality in statin users versus never users in a nested 1:2 matched study excluding individuals with aspiration pneumonia from the interstitial lung disease population.

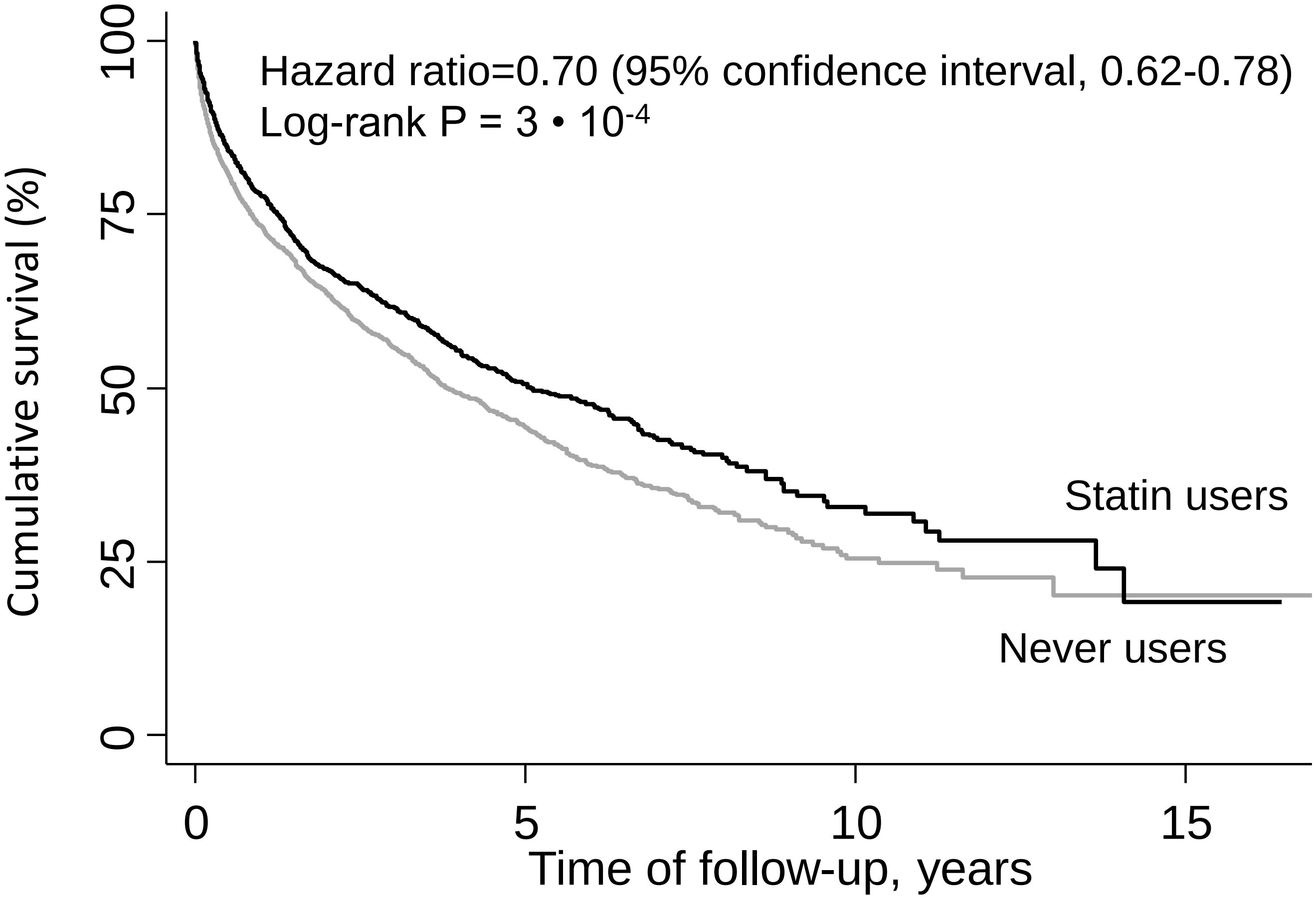

Number at risk				
Statin users	1,129	300	37	2
Never users	2,258	490	44	5
