# Supplementary material for: Statin Use Is Associated with Reduced Mortality in Patients with Interstitial Lung Disease
Source: PLoS One. 2015 Oct 16;10(10):e0140571. doi: 10.1371/journal.pone.0140571 (PMC4608706; doi:10.1371/journal.pone.0140571)
Supplement: S3 Fig — Hazard ratio is shown after multivariable adjustments. (PDF) [file pone.0140571.s003.pdf]

S3 Figure: Survival and risk of all-cause mortality in statin users versus never users among individuals diagnosed with idiopathic lung fibrosis excluding individuals with ever diagnosed rheumatic or connective tissue disease.

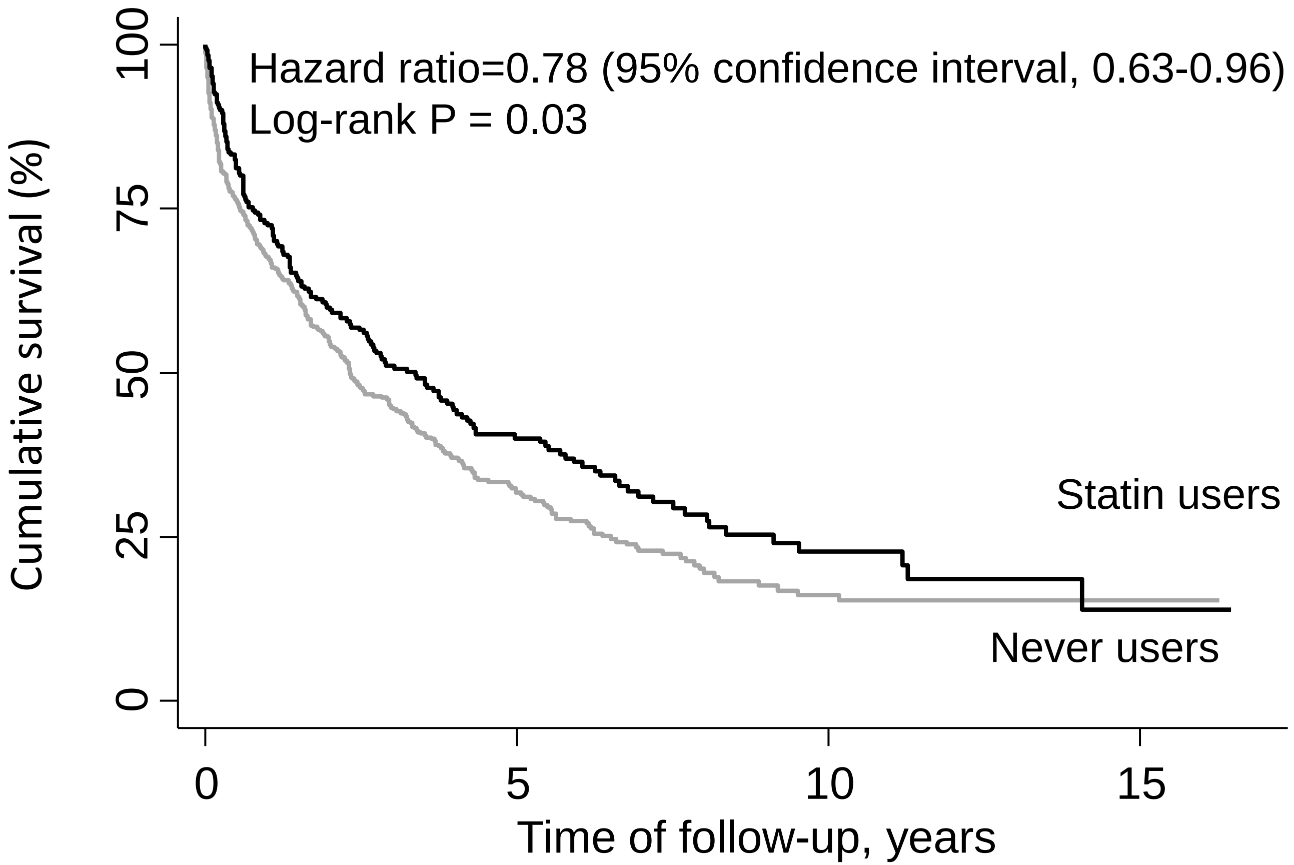

Number at risk				
Statin users	250	69	17	2
Never users	460	100	20	3
