# Supplementary material for: Statin Use Is Associated with Reduced Mortality in Patients with Interstitial Lung Disease
Source: PLoS One. 2015 Oct 16;10(10):e0140571. doi: 10.1371/journal.pone.0140571 (PMC4608706; doi:10.1371/journal.pone.0140571)

S4 Figure. Survival and risk of all-cause mortality in statin users versus never users in a nested 1:2 matched study with inclusion of patients below 40 years of age.

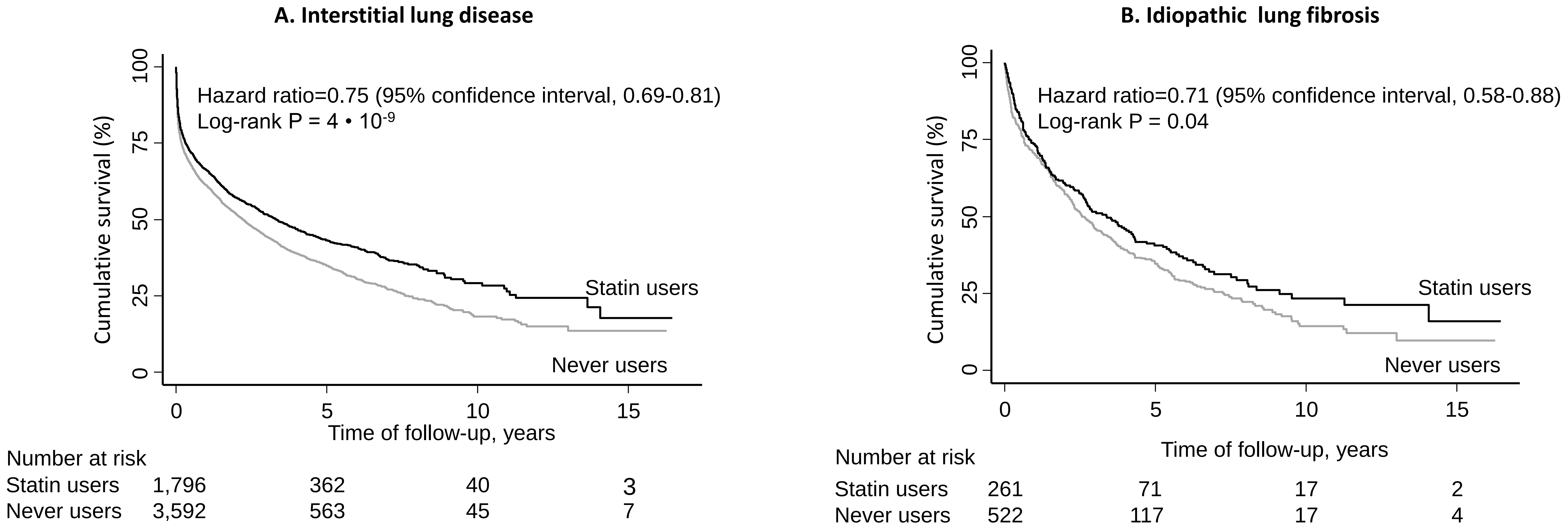

Supplement: S4 Fig — Hazard ratios are shown after multivariable adjustments. (PDF) [file pone.0140571.s004.pdf]
