# Supplementary material for: Statin Use Is Associated with Reduced Mortality in Patients with Interstitial Lung Disease
Source: PLoS One. 2015 Oct 16;10(10):e0140571. doi: 10.1371/journal.pone.0140571 (PMC4608706; doi:10.1371/journal.pone.0140571)
Supplement: S1 Table — (PDF) [file pone.0140571.s005.pdf]

S1 Table. Interstitial lung disease diagnosis codes among the patients included in the nested 1:2 matched study and from all such patients diagnosed in Denmark 1995-2009.

		**Nested 1:2 matched study**	**All patients diagnosed in Denmark 1995-2009**
**ICD-10 diagnosis**	**Sub-diagnosis of interstitial lung disease**	**Number (percent)**	**Number (percent)**
DJ60	Coalworker's lung	-	1 (0.00)
DJ609	Coalworker's pneumononiosis	3 (0.06)	35 (0.14)
DJ619	Pneumoconiosis due to asbestos and other mineral fibers	258 (4.82)	1,107 (4.15)
DJ620	Pneumoconiosis due to talc dust	1 (0.02)	14 (0.05)
DJ628	Pneumoconiosis due to other dust containing silica	20 (0.37)	180 (0.68)
DJ628A	Silicosis with lung fibrosis	2 (0.04)	4 (0.02)
DJ628B	Unspecified silicosis	4 (0.07)	11 (0.04)
DJ630	Aluminosis of lung	3 (0.06)	8 (0.03)
DJ631	Bauxite fibrosis of lung	3 (0.06)	9 (0.03)
DJ633	Graphite fibrosis of lung	2 (0.04)	13 (0.05)
DJ634	Siderosis	5 (0.05)	31 (0.12)
DJ635	Stannosis	2 (0.04)	6 (0.02)
DJ638	Pneumoconiosis due to other specified inorganic dust	9 (0.17)	42 (0.16)
DJ638A	Lung fibrosis due to stone dust	-	1 (0.00)
DJ649	Unspecified pneumoconiosis	119 (2.22)	583 (2.19)
DJ659	Pneumoconiosis associated with tuberculosis	1 (0.02)	30 (0.11)
DJ660	Byssinosis	4 (0.07)	27 (0.10)
DJ661	Flax-dresser's disease	1 (0.02)	4 (0.02)
DJ662	Cannabinosis	-	7 (0.03)
DJ668	Airway disease due to other specific organic dust	8 (0.15)	52 (0.20)
DJ670	Farmer's lung	10 (0.19)	81 (0.30)
DJ671	Bagassosis	-	4 (0.02)
DJ672	Bird fancier's lung	5 (0.09)	37 (0.14)
DJ674	Maltworker's lung	-	2 (0.01)
DJ675	Mushroom-worker's lung	-	4 (0.02)
DJ676	Maple-bark-stripper's lung	-	1 (0.00)
DJ677	Air-conditioner and humidifier lung	2 (0.04)	11 (0.04)
DJ678	Hypersensitivity pneumonitis due to other organic dusts	15 (0.28)	98 (0.37)
DJ679	Hypersensitivity pneumonitis due to unspecified organic dust	45 (0.84)	433 (1.62)
DJ680	Bronchitis and pneumonitis due to chemicals, gases, fumes, and vapours	33 (0.62)	516 (1.93)
DJ680A	Inhalation pneumonitis	2 (0.04)	2 (0.01)

DJ680B	Bronchitis due to inhaled chemicals, gases, fumes and vapours	-	20 (0.08)
DJ680C	Pneumonitis due to chemicals, gases, fumes, and vapours	1 (0.02)	20 (0.08)
DJ681	Acute pulmonary oedema due to chemicals, gases, fumes, and vapours	11 (0.21)	193 (0.72)
DJ682	Upper respiratory inflammation due to chemicals, gases, fumes, and vapours, not elsewhere classified	10 (0.19)	93 (0.35)
DJ683	Other acute and subacute respiratory conditions due to chemicals, gases, fumes, and vapours	12 (0.22)	173 (0.65)
DJ684	Chronic respiratory conditions due to chemicals, gases, fumes, and vapours	36 (0.67)	224 (0.84)
DJ684A	Lung fibrosis due to inhalation of chemicals, gases, fumes, and vapours	2 (0.04)	5 (0.02)
DJ684B	Lung emphysema due to inhalation of chemicals, gases, fumes, and vapours	1 (0.02)	1 (0.00)
DJ684C	Bronchiolitis obliterans due to chemicals, gases, fumes, and vapours	-	1 (0.00)
DJ688	Other respiratory condition due to chemicals, gases, fumes, and vapours	2 (0.04)	61 (0.23)
DJ689	Unspecified respiratory condition due to chemicals, gases, fumes, and vapours	25 (0.47)	363 (1.36)
DJ69	Pneumonitis due to solids and liquids	-	7 (0.03)
DJ690	Pneumonitis due to food and vomit	1,447 (27.01)	5,569 (20.89)
DJ691	Pneumonitis due to oils and essences	14 (0.26)	99 (0.37)
DJ698	Pneumonitis due to other solids and liquids	327 (6.10)	1,457 (5.47)
DJ698A	Pneumonitis due to blood	9 (0.17)	40 (0.15)
DJ70	Respiratory conditions due to other external agents	-	1 (0.00)
DJ700	Acute pulmonary manifestations due to radiation	23 (0.43)	69 (0.26)
DJ701	Chronic and other pulmonary manifestations due to radiation	32 (0.60)	173 (0.65)
DJ702	Acute drug-induced interstitial lung	18 (0.36)	61 (0.23)

	disorders		
DJ703	Chronic drug-induced interstitial lung disorder	11 (0.21)	35 (0.13)
DJ704	Drug-induced interstitial lung disorder, unspecified	40 (0.75)	202 (0.76)
DJ708	Respiratory conditions due to other specified external agents	5 (0.09)	45 (0.17)
DJ709	Respiratory conditions due to unspecified external agent	44 (0.82)	286 (1.07)
DJ84	Other interstitial pulmonary diseases	-	8 (0.03)
DJ840	Alveolar and perialveolar conditions	19 (0.35)	93 (0.35)
DJ841	Other interstitial pulmonary diseases with fibrosis (idiopathic pulmonary fibrosis)	706 (13.18)	5,840 (21.90)
DJ841A	Idiopathic pulmonary fibrosis with usual interstitial pneumonia (UIP)	9 (0.17)	67 (0.25)
DJ841B	Idiopathic pulmonary fibrosis with non-specific interstitial pneumonia (NSIP)	43 (0.80)	215 (0.81)
DJ841C	Idiopathic pulmonary fibrosis with interstitial pneumonia	14 (0.26)	71 (0.27)
DJ841D	Acute interstitial pneumonitis (AIP)	2 (0.04)	23 (0.09)
DJ841E	Idiopathic pulmonary fibrosis	9 (0.17)	46 (0.17)
DJ842	Desquamative interstitial pneumonia (DIP)	4 (0.07)	8 (0.03)
DJ843	Bronchiolitis obliterans organizing pneumonia (BOOP)	16 (0.30)	53 (0.20)
DJ848	Other specified interstitial pulmonary diseases	232 (4.33)	841 (3.15)
DJ848A	Allergic pulmonary aspergillosis	2 (0.04)	6 (0.02)
DJ849	Interstitial pulmonary disease, unspecified	1,675 (31.26)	6,836 (25.64)
**Total**		**5,358 (100.00)**	**26,659 (100.00)**
